# IL‐6 Exacerbates Experimental Autoimmune Prostatitis by Disrupting STAT5a‐Mediated Treg Cell Function and Th17/Treg Balance

**DOI:** 10.1155/mi/2600849

**Published:** 2026-05-26

**Authors:** Xianhong Liu, Xiaokang Bian, Shanchuan Han, Boyang Li, Jian Song, Chaozhao Liang, Jun Zhou, Xianguo Chen

**Affiliations:** ^1^ Department of Urology, The First Affiliated Hospital of Anhui Medical University, Hefei, China, ahmu.edu.cn; ^2^ Institute of Urology, Anhui Medical University, Hefei, China, ahmu.edu.cn; ^3^ Anhui Province Key Laboratory of Urological and Andrological Diseases Research and Medical Transformation, Hefei, China; ^4^ Department of Urology, East City Campus of First Affiliated Hospital of Anhui Medical University, Hefei, China

**Keywords:** chronic prostatitis/chronic pelvic pain syndrome, IL-6, STAT5a, Treg

## Abstract

**Objective:**

Chronic prostatitis/chronic pelvic pain syndrome (CP/CPPS) is a common urological condition in men. Although disruption of the Th17 (T helper 17)/Treg (regulatory T) balance has been implicated in its pathogenesis, the upstream drivers of this immune imbalance remain incompletely understood. This study investigated whether interleukin‐6 (IL‐6) is associated with altered Th17/Treg homeostasis and impaired Treg function in experimental autoimmune prostatitis (EAP).

**Methods:**

An EAP model was generated in NOD mice through immunization with prostate antigen. Th17/Treg cell profiles were determined by flow cytometry, cytokine concentrations by enzyme‐linked immunosorbent assay (ELISA), and signal transducer and activator of transcription 5a (STAT5a) expression by western blotting and immunohistochemistry (IHC). In vivo IL‐6 inhibition was used to assess its effects on Th17/Treg balance and Treg suppressive function, whereas in vitro experiments examined the effects of IL‐6 on Treg function and naïve CD4^+^ T‐cell differentiation.

**Results:**

Relative to control mice, EAP mice showed higher serum IL‐6 concentrations (5.28 ± 0.54 vs. 3.36 ± 0.46 pg/mL, *p* < 0.01), together with disturbed Th17/Treg homeostasis, as reflected by expansion of Th17 cells (1.72% ± 0.18% vs. 0.98% ± 0.08%, *p* < 0.001) and reduction of Treg cells (3.99% ± 0.97% vs. 5.46 % ± 0.53%, *p* < 0.05). Suppressive activity of Treg cells was also diminished (6.56% ± 0.80% vs. 12.41% ± 3.49%, *p* < 0.05). In vitro, IL‐6 shifted naïve CD4^+^ T‐cell differentiation toward the Th17 phenotype while limiting Treg generation and impairing Treg suppressive activity. Mechanistically, IL‐6 stimulation reduced STAT5a and p‐STAT5a expression in Treg cells. Pharmacological inhibition of IL‐6 partially restored Treg function and alleviated inflammatory pathology in EAP mice.

**Conclusion:**

IL‐6 is linked to Th17/Treg imbalance and Treg dysfunction in EAP, possibly through inhibition of STAT5a signaling, and IL‐6‐related pathways may offer therapeutic value for CP/CPPS.

## 1. Introduction

Chronic prostatitis/chronic pelvic pain syndrome (CP/CPPS) is a frequently encountered urologic disorder that primarily affects young and middle‐aged men, with an estimated worldwide prevalence of 8.4%–14% [1]. Clinically, it is mainly characterized by chronic pelvic pain and lower urinary tract symptoms, including urgency, frequency, and dysuria [2]. The disorder substantially compromises patients’ quality of life and contributes to a considerable socioeconomic burden [3, 4]. Nevertheless, the etiology and pathogenic mechanisms of CP/CPPS remain to be fully elucidated. Current evidence indicates that chronic inflammation and immune dysregulation play important roles in the onset and persistence of CP/CPPS. In particular, aberrant T‐cell responses within the prostate microenvironment have been proposed as central drivers of sustained inflammatory reactions in CP/CPPS [5].

The experimental autoimmune prostatitis (EAP) model has been extensively applied to explore the immunopathogenesis of CP/CPPS, as it recapitulates several key features of the human disease, including prostatic lymphocytic infiltration, chronic inflammation, and pain‐related behaviors [6–8]. Among the immune components involved, T helper 17 (Th17) cells are recognized as key contributors to inflammation‐related tissue damage. Th17 cells are distinguished by the expression of retinoic acid receptor‐related orphan receptor gamma t (RORγt) and their ability to produce pro‐inflammatory cytokines, especially interleukin‐17 (IL‐17), which promotes neutrophil recruitment and amplifies local inflammatory responses [9]. Previous EAP studies have shown increased infiltration of Th17 cells in inflamed prostate tissue, and blockade of IL‐17 signaling significantly attenuates prostatic inflammation, supporting a pathogenic role for Th17‐mediated immune responses in prostatitis [7, 10]. In contrast, regulatory T (Treg) cells are critical for maintaining immune tolerance and restraining excessive immune activation. They restrain effector T‐cell responses through several pathways, including the release of anti‐inflammatory cytokines and direct cell–cell contact [11]. Maintenance of the Th17/Treg balance is therefore critical for immune homeostasis. However, accumulating evidence indicates that disruption of this balance represents a critical immunological feature of CP/CPPS. Both clinical studies and experimental investigations have reported increased Th17 cell frequencies accompanied by reduced Treg proportions in CP/CPPS patients and EAP mice [12, 13]. Although these findings suggest that Th17/Treg imbalance contributes to disease progression, emerging studies indicate that alterations in Treg suppressive function may be equally important in sustaining chronic inflammation. Indeed, impaired Treg function has been observed in EAP models and is thought to contribute to persistent inflammatory responses within the prostate [14]. Nevertheless, the molecular mechanisms responsible for Treg functional instability in prostatitis remain poorly defined. Although both clinical CP/CPPS studies and EAP investigations consistently support a role for Th17/Treg disequilibrium, most previous work has focused primarily on changes in cell proportions rather than the mechanisms governing Treg functional stability. In addition, while EAP models have provided mechanistic evidence for immune‐mediated prostatic inflammation, the upstream inflammatory signals linking the prostatitis microenvironment to Treg dysfunction remain insufficiently defined.

Interleukin‐6 (IL‐6) is a multifunctional mediator that exerts broad regulatory effects on immune and inflammatory responses. Increased IL‐6 levels have been documented in multiple chronic inflammatory disorders and are frequently linked to disease severity and progression [15–17]. Notably, elevated IL‐6 expression has also been observed in patients with prostatitis and in experimental models of prostatic inflammation [18, 19]. As an important modulator of T‐cell differentiation, IL‐6 favors the polarization of naïve CD4^+^ T‐cells toward the Th17 lineage while suppressing their differentiation into Treg cells, thereby disturbing immune homeostasis [20, 21]. Although the role of IL‐6 in T‐cell polarization has been extensively investigated, its potential contribution to the maintenance of Treg functional stability in prostatitis remains insufficiently defined. Signal transducer and activator of transcription 5a (STAT5a) signaling is required for the development and maintenance of Treg cells. Activation of this pathway facilitates the differentiation of naive CD4^+^ T‐cells into the Treg lineage and supports their suppressive function and lineage stability [22]. Conversely, disruption of STAT5a signaling can compromise Treg function and facilitate the development of immune‐mediated inflammatory disorders. However, whether IL‐6 interferes with STAT5a signaling in Treg cells within the inflammatory microenvironment of prostatitis remains unclear. Clarifying how inflammatory cytokines affect Treg stability may improve understanding of the mechanisms underlying immune dysregulation in CP/CPPS.

Accordingly, this study aimed to examine the involvement of IL‐6 in Treg function and Th17/Treg homeostasis in EAP. Specifically, we sought to determine whether IL‐6 contributes to Treg dysfunction through modulation of STAT5a signaling. We further hypothesized that elevated IL‐6 levels within the inflammatory microenvironment may impair Treg suppressive activity and disturb the Th17/Treg balance, thereby exacerbating prostatitis.

## 2. Materials and Methods

### 2.1. Reagents and Antibodies

Complete Freund’s Adjuvant (CFA; Sigma–Aldrich); IL‐17 (cat# EK0431; Boster Biotechnology); IL‐6 (cat# EK0411; Boster Biotechnology); Recombinant Interleukin‐6 (cat# CG39; Novoprotein); Recombinant Interleukin‐2 (cat# CK24); Recombinant Human Transforming Growth Factor‐β (cat# CA59; Novoprotein); Anti‐CD3 Antibody (cat# BE0001‐1; Bio X Cell); Anti‐CD28 Antibody (cat# BE0015‐1; Bio X Cell); Anti‐Mouse CD4‐FITC (cat# 553047; BD Biosciences); Anti‐Mouse CTLA‐4‐APC (cat# 106309; Biolegend); Anti‐Mouse Foxp3‐Violet 421 (cat# 126419; Biolegend); Anti‐Mouse IL‐17 A‐Violet 421 (cat# 563354; BD Biosciences); Anti‐Mouse Foxp3‐PE (cat# 563101; BD Biosciences); Anti‐GAPDH (cat# AF7021; Affinity); Anti‐STAT5a (cat# AF6303; Affinity); Anti‐Phospho‐STAT5a (cat# AF3303; Affinity); PMA (50601ES03; Yeasen); Ionomycin (50401ES03; Yeasen); Manganese Ionomycin (cat# 50501ES60); Fixation and Permeabilization Buffer (8222‐49 and 8333‐56, eBioscience); LMT‐28 (cat# HY‐102084); Stafia‐1 (cat# GC62559).

### 2.2. Murine Model Establishment

Male NOD mice (5 weeks old, 18 ± 2 g) were purchased from the Nanjing Institute of Biomedical Research, Nanjing University (Nanjing, China). Twenty mice were included and housed under specific pathogen‐free conditions. In the first cohort, mice were assigned to either the control group (*n* = 4) or the EAP group (*n* = 4). In the second cohort, mice were distributed into three groups: control (*n* = 4), EAP (*n* = 4), and EAP treated with LMT‐28 (*n* = 4). All in vivo procedures were performed in accordance with approval granted by the Institutional Animal Care and Use Committee of Anhui Medical University (Approval Number LLSC20211521).

### 2.3. Development, Classification, and Pain Assessment in the Mouse Model

Prostate antigen was prepared by homogenizing prostate tissue obtained from SD rats and emulsifying it with an equal volume of CFA to generate an immunogenic preparation [23]. A subcutaneous injection of 0.15 mL of the immunogenic reagent (300 mg per mouse) was administered on days 0 and 15 at three sites: 0.050 mL in the shoulder, 0.050 mL at the tail’s base, and 0.025 mL in each footpad. The control cohort was administered 0.9% sterile saline solution (vehicle) in lieu of prostate antigen immunization, with terminal procedures performed at the 28‐day experimental endpoint. Upon euthanasia, the footpads of the mice appeared pink, and no abnormal behaviors were observed during walking or standing. Starting from day 16, LMT‐28 (an IL‐6 receptor inhibitor) was dissolved in sterile corn oil and administered daily by gavage at a dose of 1 mg/kg for 14 days until the end of the experiment. Two additional groups of mice received the same volume of sterile corn oil each day. On day 28, the frequency of responses to pelvic stimulation was recorded. Positive pain responses were identified as (a) pronounced contraction of the abdominal muscles, (b) immediate licking or scratching at the stimulated site, or (c) jumping.

### 2.4. Enzyme‐Linked Immunosorbent Assay (ELISA), Immunohistochemical Analysis, and H&E Staining

Prostate samples were fixed in paraformaldehyde, processed through graded ethanol for dehydration, and then embedded in paraffin. Consecutive 4 μm sections were prepared for histological and immunohistochemical evaluation. Prostatic inflammation was assessed by hematoxylin–eosin (H&E) staining using a semiquantitative scoring system as previously described [24]. Inflammatory infiltration was evaluated using a 4‐point scale (0–3): 0 indicated absence of inflammatory cell infiltration; 1 indicated slight infiltration characterized by scattered mononuclear cells; 2 indicated intermediate infiltration with focal inflammatory aggregates and perivascular cuffing; and 3 indicated marked inflammation with widespread inflammatory cell infiltration and extensive involvement of the prostatic parenchyma. For immunohistochemistry (IHC) analysis, deparaffinized tissue sections underwent antigen retrieval in ethylenediaminetetraacetic acid (EDTA) buffer, endogenous peroxidase blocking, and serum blocking, followed by incubation with anti‐STAT5a antibody (1:800, #AF6303, Affinity) and secondary antibody (1:200, Servicebio). Digital images were acquired using a Pannoramic MIDI scanner. Commercial ELISA kits were used to quantify serum IL‐10, IL‐17, and IL‐6 levels in accordance with the manufacturers’ instructions.

### 2.5. Western Blotting

Treg cells were lysed with radioimmunoprecipitation assay (RIPA) buffer for analysis of p‐STAT5a and STAT5a protein expression. Proteins were resolved on 12.5% sodium dodecyl sulfate (SDS)‐polyacrylamide gels and subsequently transferred onto nitrocellulose membranes. Glyceraldehyde‐3‐phosphate dehydrogenase (GAPDH) served as the internal loading reference. The membranes were incubated with primary antibodies (1:1000), followed by horseradish peroxidase (HRP)‐linked secondary antibodies (1:5000), and the immunoreactive signals were visualized by enhanced chemiluminescence.

### 2.6. Naïve CD4^+^ T‐Cell Isolation and Differentiation

Mouse splenocytes were used to obtain naïve CD4^+^ T‐cells for in vitro induction based on our previously established method [25]. After magnetic isolation with a commercial kit (Miltenyi Biotec, #130‐104‐453), cells were seeded into anti‐CD3/CD28‐coated 24‐well plates at a density of 1 × 10^5^ cells per well and maintained in RPMI‐1640 medium containing 10% fetal bovine serum (FBS) and 1% antibiotics. To induce differentiation, cells were cultured with IL‐6 (40 ng/mL), IL‐2 (20 ng/mL), TGF‐β1 (1 ng/mL), anti‐IFN‐γ (20 μg/mL), and anti‐IL‐4 (20 μg/mL), whereas the control group was maintained under the same conditions without IL‐6. Cells were collected on day 4 for subsequent analyses.

### 2.7. Sorting of Cells

To obtain CD4^+^CD25^+^ cells, mouse splenocytes were incubated for 40 min at 4°C with fluorescent anti‐CD4 and anti‐CD25 antibodies, followed by sorting of the labeled population. The isolated cells showed a purity above 95%.

### 2.8. Treg Cell Suppression Assay

Splenic Teff and Treg cells were prepared from control mice. Carboxyfluorescein succinimidyl ester (CFSE)‐stained CD4^+^CD25^-^Teff cells were mixed with CD4^+^CD25^+^Treg cells at a 1:1 ratio and maintained in anti‐CD3/CD28‐coated 24‐well plates containing RPMI‐1640 medium with 10% FBS, 1% antibiotics, and 20 ng/mL IL‐2. After 72 h, cell proliferation was evaluated by flow cytometry according to CFSE dilution and processed with FlowJo software.

### 2.9. Statistics

All datasets were generated from no fewer than three independent experimental repeats. Data are expressed as mean ±standard deviation (SD), and all analyses were performed using GraphPad Prism 9. Significance between two conditions was tested with Student’s *t*‐test, whereas comparisons across more than two conditions were assessed by one‐way analysis of variance (ANOVA). Results were considered significant when *p* was below 0.05.

## 3. Results

### 3.1. Th17/Treg Imbalance and Impaired Treg Suppressive Function in EAP Mice

**Figure 1:**
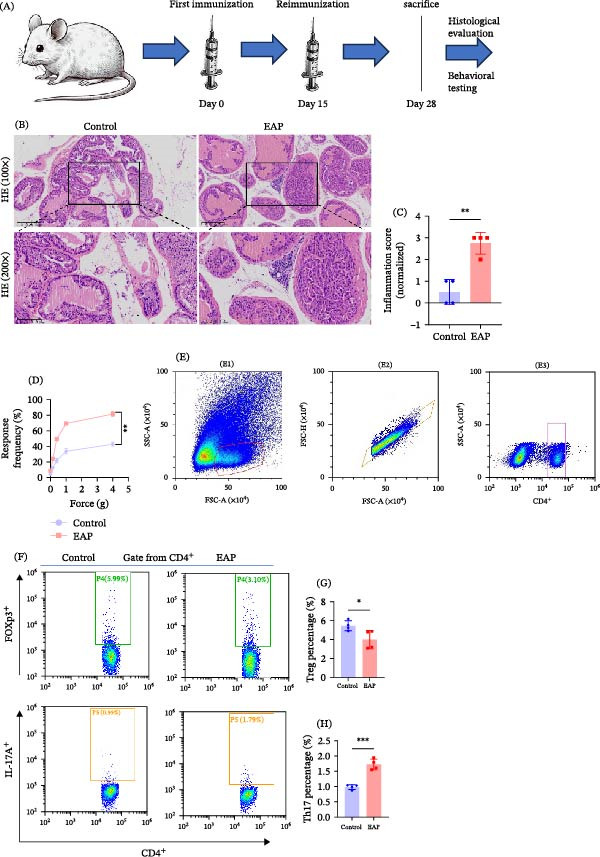
Evaluation of the EAP model and Th17/Treg imbalance. (A) Immunization schedule for age‐matched mice on day 0 and day 15. EAP was established after 2 weeks, and pain was assessed 1 day before sacrifice. Animals were euthanized on day 28, and tissue samples were harvested. (B) Representative H&E‐stained prostate sections from control and EAP mice. (C) Prostatic inflammation scores in the control and EAP groups. *n* = 4 per group. Group comparisons were analyzed using Student’s *t*‐test. (D) Mechanical pain responses assessed by von Frey filaments in both groups. *n* = 4 per group. Student’s *t*‐test was used for group comparisons. (E) Gating strategy for flow cytometry analysis including (E1) lymphocyte gating based on FSC‐A and SSC‐A, (E2) singlet gating based on FSC‐A and FSC‐H, and (E3) CD4+ T‐cell gating. (F–H) Flow cytometry analysis of the percentages of CD4^+^Foxp3^+^ (Treg) and CD4^+^IL‐17A^+^ (Th17) cells in the spleens of both groups. *n* = 4 per group. Differences between groups were analyzed with Student’s *t*‐test.  ^∗^ *p* < 0.05;  ^∗∗^ *p* < 0.01;  ^∗∗∗^ *p* < 0.001. H&E, hematoxylin–eosin staining.

**Figure 2:**
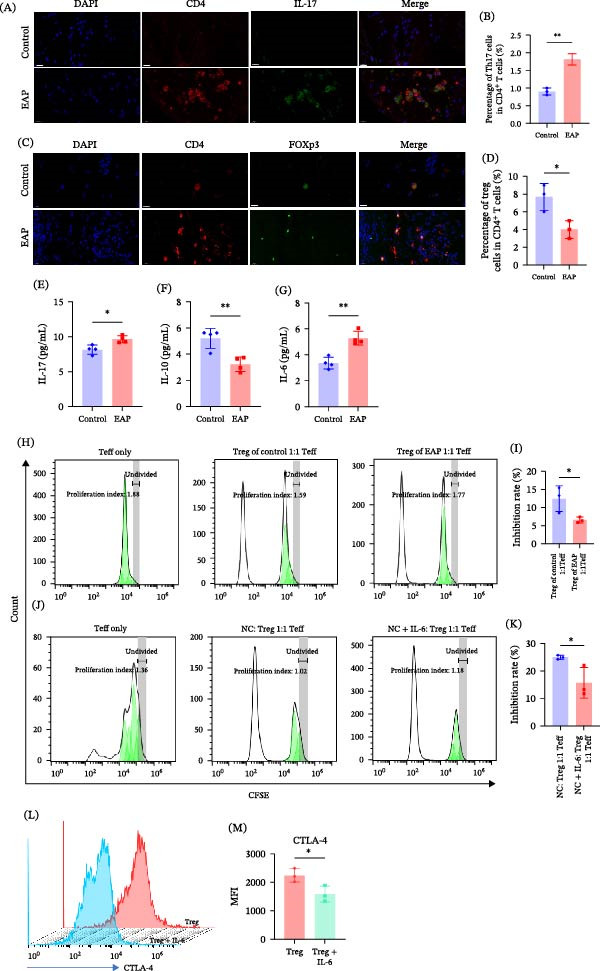
Dysregulation of Th17/Treg balance with concomitant Treg dysfunction in prostate tissues. (A) Representative immunofluorescence images showing IL‐17 and CD4 expression in prostate sections from control and EAP mice. (B) Quantitative analysis of Th17 cell counts in immunofluorescence sections between groups (*n* = 3). Group comparisons were conducted using Student’s *t*‐test. (C) Representative immunofluorescence staining of Foxp3 and CD4 in prostate tissues from the control and EAP groups. (D) Treg cell numbers were quantified in immunofluorescence‐stained sections from the two groups (*n* = 3). Comparisons between groups were performed with Student’s *t*‐test. (E–G) Serum concentrations of IL‐6, IL‐17, and IL‐10 were measured by ELISA (*n* = 4). Differences were analyzed using Student’s *t*‐test. (H) Control‐ or EAP‐derived purified Treg cells were incubated with carboxyfluorescein succinimidyl ester (CFSE)‐labeled effector T‐cells at an equal ratio for 3 days, and cell proliferation was quantified by flow cytometry according to dye dilution. Proliferation curves were fitted using FlowJo software. Inhibition rate calculated using formula: (1− [Tregs:teff 1:1 PI]/[Tregs:teff 0:1 PI]) × 100%. Representative proliferation kinetics shown. (I) Inhibition rates of Treg cells from both groups on Teff cell proliferation (*n* = 3). Statistical significance was assessed using Student’s *t*‐test. (J) Control Treg cells partitioned into NC (IL‐2 alone, 20 ng/mL) and NC + IL‐6 groups (IL‐2 + IL‐6, 80 ng/mL). Cocultured with CFSE‐labeled Teff cells at 1:1 ratio for 3 days. CFSE proliferation signals analyzed via flow cytometry. Proliferation curves were fitted using FlowJo software. Inhibition rate calculated using formula: (1 − [Tregs:teff 1:1 PI]/[Tregs:teff 0:1 PI]) × 100%. Representative proliferation kinetics shown. (K) Inhibition rates of Treg cells from both groups on Teff cell proliferation (*n* = 3). Statistical significance was assessed using Student’s *t*‐test. (L) Control Treg cells were partitioned into NC (IL‐2 alone, 20 ng/mL) and NC + IL‐6 groups (IL‐2, 20 ng/mL, IL‐6, 80 ng/mL). Cells were cultured for 48 h under respective stimulations. CTLA‐4 expression detected by flow cytometry. Representative images shown. (M) Quantitative analysis of CTLA‐4 MFI between groups (*n* = 3). Statistical significance was assessed using Student’s *t*‐test.  ^∗^ *p* < 0.05;  ^∗∗^ *p* < 0.01; PI, proliferation index; MFI, mean fluorescence intensity.

To establish the EAP model, mice received immunization with prostate antigens emulsified with CFA (Figure 1A). Histological examination of prostate tissues revealed marked lymphocytic infiltration in the EAP group relative to controls (Figure 1B). Quantitative histological scoring confirmed a significant increase in inflammation in EAP mice relative to control mice (*p* < 0.01; Figure 1C). Behavioral testing further showed that EAP mice had significantly greater pelvic pain responses than control mice (Figure 1D). Analysis of splenic CD4^+^ T‐cells by flow cytometry revealed a clear imbalance in T‐cell subset distribution in EAP mice. Compared with control animals, EAP mice displayed a lower proportion of Treg cells (3.99% ± 0.97% vs. 5.46% ± 0.53%, *p* < 0.05; Figure 1F,G) but a higher proportion of Th17 cells (1.72% ± 0.18% vs. 0.98 % ± 0.08%, *p* < 0.001; Figure 1F–H). Consistently, immunofluorescence staining of prostate tissues showed markedly greater Th17 cell infiltration (*p* < 0.01; Figure 2A,B), accompanied by a reduction in Treg cells (*p* < 0.05; Figure 2C,D). Serum cytokine analysis showed that IL‐17 levels were significantly elevated (9.64 ± 0.5 vs. 8.15 ± 0.66 pg/mL, *p* < 0.05; Figure 2E), whereas IL‐10 levels were significantly reduced (3.23 ± 0.53 vs. 5.45 ± 0.75 pg/mL, *p* < 0.01; Figure 2F). Additionally, circulating IL‐6 levels were significantly increased in EAP mice (5.28 ± 0.54 vs. 3.36 ± 0.46 pg/mL, *p* < 0.01; Figure 2G). Functional suppression assays further demonstrated that Treg cells isolated from EAP mice exhibited significantly reduced suppressive activity compared with control Treg cells (6.56% ± 0.8% vs. 12.41% ± 3.49%, *p* < 0.05; Figure 2H,I).

### 3.2. IL‐6 Weakens Treg Suppressive Capacity

Given the elevated IL‐6 levels observed in EAP mice, we next investigated whether IL‐6 directly influences Treg function. In vitro stimulation with IL‐6 significantly reduced the suppressive capacity of Treg cells in the Treg–Teff coculture system. The inhibition rate decreased from 25.00% ± 0.74% in control Treg cells to 15.69% ± 5.57% following IL‐6 treatment (*p* < 0.05; Figure 2J,K). Flow cytometry analysis further demonstrated that IL‐6 stimulation significantly reduced the expression of cytotoxic T‐lymphocyte‐associated protein 4 (CTLA‐4), an important molecule mediating Treg suppressive function. The mean fluorescence intensity (MFI) of CTLA‐4 decreased from 2245.67 ± 234.64 to 1592.33 ± 279.83 (*p* < 0.05; Figure 2L,M).These findings indicate that IL‐6 may impair the suppressive function of Treg cells.

### 3.3. IL‐6 Disrupts Th17/Treg Balance Through Suppression of STAT5a Signaling

**Figure 3:**
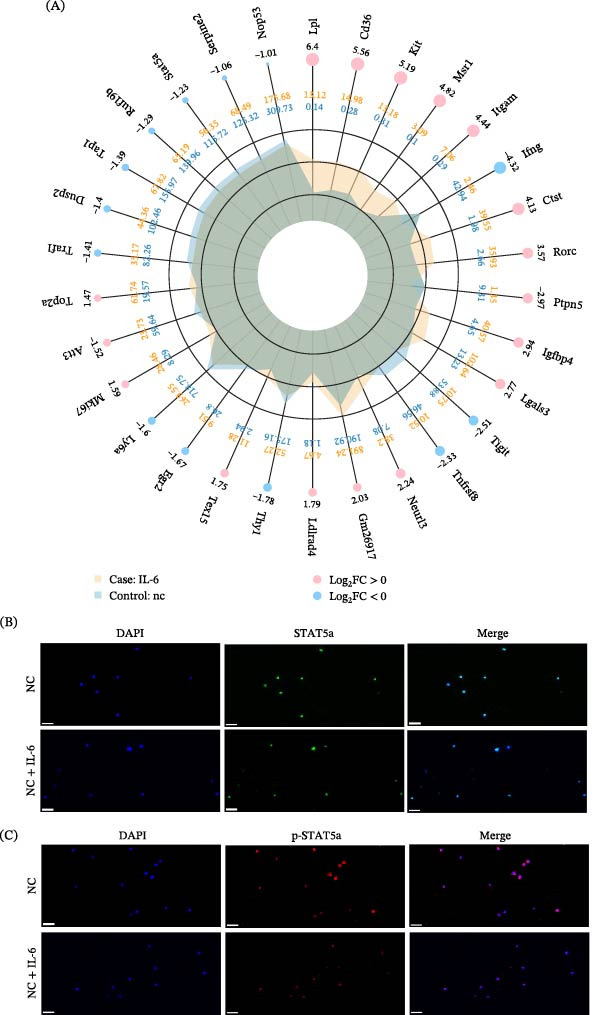
IL‐6 suppresses STAT5a signaling in regulatory T‐cells: transcriptional and immunofluorescence evidence of STAT5a/p‐STAT5a downregulation. (A) Radar plot comparing gene expression patterns between IL‐6‐stimulated (case: IL‐6) and control (nc) groups. Axes represent individual genes, with data points indicating log_2_ fold changes (log_2_FC). Upregulated genes (pink) and downregulated genes (blue) are differentially colored. Notably, STAT5a exhibits significant downregulation in the IL‐6 group. (B) Immunofluorescence analysis reveals substantially reduced STAT5a protein expression in IL‐6‐stimulated Treg cells compared to control groups. (C) Phosphorylated STAT5a (p‐STAT5a) immunofluorescence demonstrates marked attenuation in IL‐6‐treated Treg populations relative to untreated controls.

**Figure 4:**
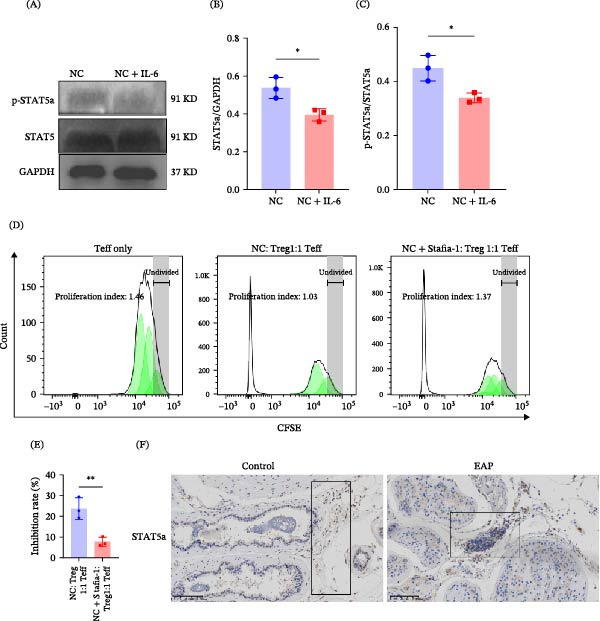
IL‐6 impairs Treg suppressive function via STAT5a downregulation. (A) Protein levels in purified Treg cells exposed or not exposed to IL‐6 were assessed by immunoblotting. (B) Relative grayscale values of STAT5a bands normalized to GAPDH. (C) Relative grayscale analysis of p‐STAT5a bands normalized to total STAT5a. (D) Purified Treg cells were assigned to two conditions, one of which received the STAT5a inhibitor Stafia‐1. The two groups were then cocultured with carboxyfluorescein succinimidyl ester (CFSE)‐labeled effector T‐cells (Teffs) at a 1:1 ratio for 3 days. CFSE fluorescence was quantified by flow cytometry, and the results were processed using FlowJo software. The suppression rate was calculated as: (1 − [Tregs:Teffs‐1:1 PI]/[Tregs:Teffs 0:1 PI]) × 100%. Representative proliferation kinetics shown. (E) Suppression rates of Treg cells and STAT5a inhibitor‐treated Treg cells on Teff proliferation. (F) Representative immunohistochemical staining of STAT5a in prostate sections from control and EAP mice.  ^∗^ *p* < 0.05;  ^∗∗^ *p* < 0.01.

**Figure 5:**
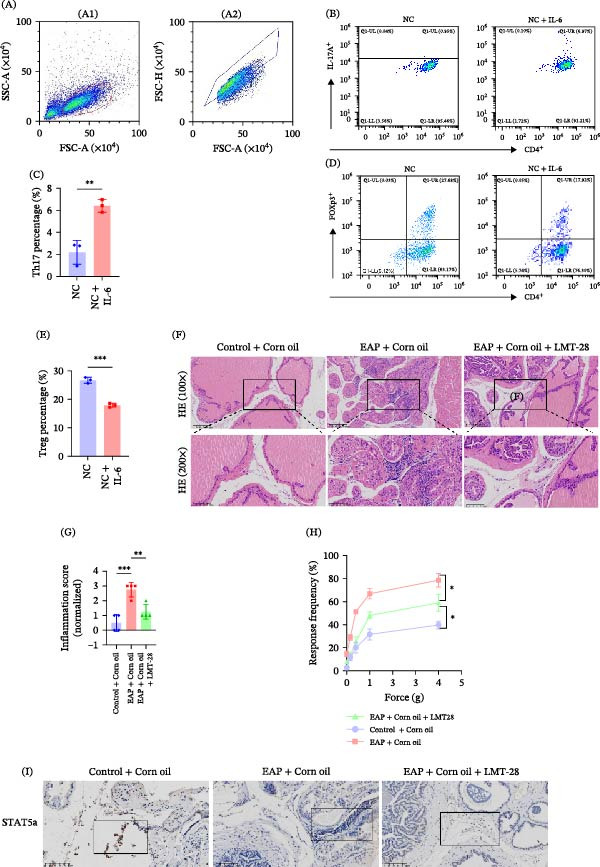
IL‐6 Drives Th17/Treg Imbalance and Inflammation in EAP. (A) Flow cytometry‐based gating approach for analyzing naïve CD4^+^ T‐cell differentiation in vitro including (A1) selection of the main cell population and exclusion of debris based on FSC‐A and SSC‐A and (A2) singlet gating based on FSC‐A and FSC‐H. (B) Flow cytometry plots showing Th17 (CD4^+^IL‐17A^+^ T) cells in the NC and NC + IL‐6 groups. (C) Proportions of Th17 cells in both groups. (D) Representative flow cytometry plots of Treg cells (CD4^+^Foxp3^+^ T) from the two groups. (E) Relative proportions of Treg cells in both groups. (F) Representative H&E staining images of prostate tissues from the three groups. (G) Histological inflammation scores of prostate tissues among the three groups (*n* = 4). Intergroup comparisons were analyzed by ANOVA. (H) Assessment of abnormal pain responses using von Frey filaments in the three groups (*n* = 4). Intergroup comparisons were analyzed by ANOVA. (I) Immunohistochemical detection of STAT5a in prostate tissues obtained from the three groups.  ^∗^ *p* < 0.05;  ^∗∗^ *p* < 0.01;  ^∗∗∗^ *p* < 0.001.

To investigate the molecular mechanism underlying IL‐6‐mediated Treg dysfunction, transcriptome sequencing was performed in IL‐6‐treated Treg cells. RNA sequencing (RNA‐seq) analysis revealed that STAT5a expression was significantly downregulated following IL‐6 stimulation (Figure 3A). Immunofluorescence staining confirmed that IL‐6 treatment markedly reduced STAT5a expression in Treg cells (Figure 3B) and attenuated the p‐STAT5a immunofluorescence signal (Figure 3C). Western blot analysis further demonstrated that IL‐6 significantly decreased both total STAT5a and phosphorylated STAT5a (p‐STAT5a) levels (*p* < 0.05; Figure 4A–C). To determine whether STAT5a signaling regulates Treg suppressive function, Treg cells were treated with the STAT5a inhibitor Stafia‐1. Inhibition of STAT5a significantly reduced Treg suppressive activity (7.76% ± 2.01% vs. 23.74% ± 5.24%, *p* < 0.01; Figure 4D,E). Furthermore, immunohistochemical staining of prostate tissues revealed reduced STAT5a expression in infiltrating lymphocytes in the EAP group relative to controls (Figure 4F). To clarify the impact of IL‐6 on T‐cell lineage commitment, naïve CD4^+^ T‐cells were cultured in a polarization system in the presence of IL‐6. Flow cytometric analysis demonstrated that IL‐6 significantly inhibited Treg differentiation (17.92% ± 0.70% vs. 26.72% ± 1.02%, *p* < 0.001; Figure 5D,E) while promoting Th17 cell differentiation (6.41% ± 0.57% vs. 2.19% ± 1.08%, *p* < 0.01; Figure 5B,C).

### 3.4. Inhibition of IL‐6 Restores Treg Function and Alleviates Th17/Treg Imbalance

**Figure 6:**
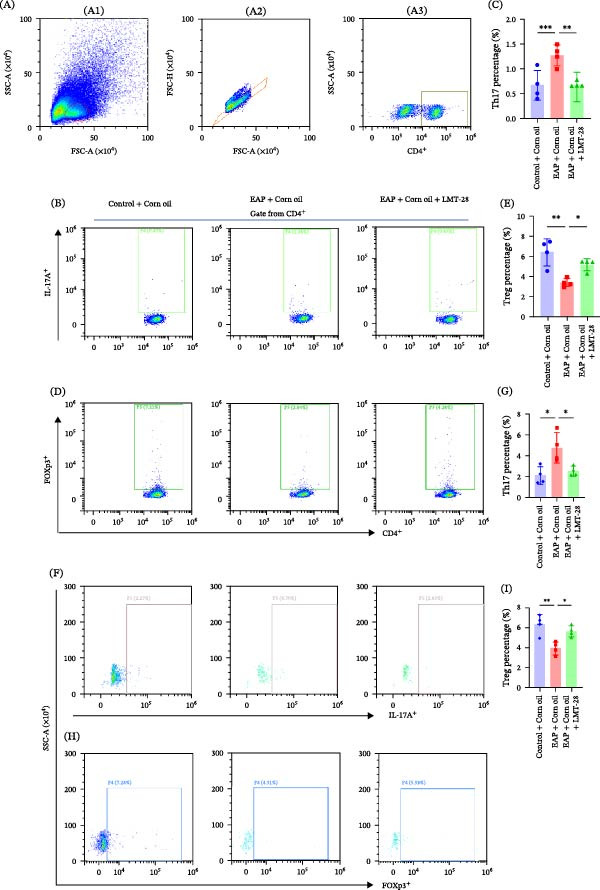
Flow cytometry analysis of Th17 and Treg cell proportions in spleen and prostate tissues. (A) Flow cytometric gating scheme, including (A1) lymphocyte gating based on FSC‐A and SSC‐A, (A2) singlet gating based on FSC‐A and FSC‐H, and (A3) CD4^+^ T‐cell gating. (B) Flow cytometry plots showing Th17 cells in splenocytes from the three groups. (C) Percentages of Th17 cells in splenocytes from the three groups (*n* = 4). Intergroup comparisons were analyzed by ANOVA. (D) Flow cytometry plots showing Treg cells in splenocytes from the three groups. (E) Treg cell frequencies in splenocytes from the three groups (*n* = 4). Intergroup comparisons were analyzed by ANOVA. (F) Flow cytometry plots showing Th17 cells in prostate sections from the three groups. (G) Quantification of Th17 cell percentages in prostate tissues from the three groups (*n* = 4). Intergroup comparisons were analyzed by ANOVA. (H) Flow cytometric profiles of Treg cells in prostate tissues from all three groups. (I) Relative proportions of Treg cells in prostate tissues among the three groups (*n* = 4). Statistical differences were determined by analysis of variance (ANOVA).  ^∗^ *p* < 0.05;  ^∗∗^ *p* < 0.01.

**Figure 7:**
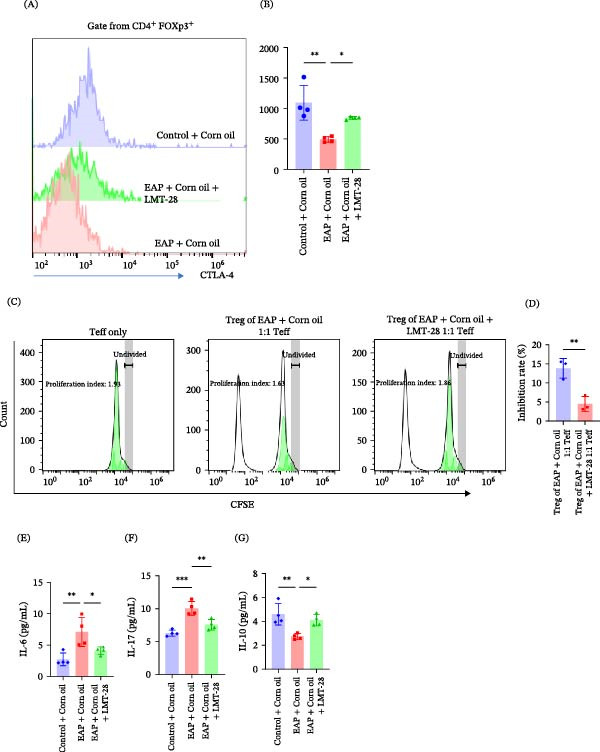
IL‐6 Inhibition restores treg function and alters cytokine levels in EAP. (A) Flow cytometry analysis of CTLA‐4 expression in Treg cells from splenocytes of the three groups. (B) CTLA‐4 fluorescence intensity in splenic Treg cells among the three groups (*n* = 4). Intergroup comparisons were analyzed by ANOVA. (C) Treg cells purified from EAP + corn oil and EAP + corn oil + LMT‐28 groups were incubated with carboxyfluorescein succinimidyl ester (CFSE)‐labeled effector T‐cells (Teffs) at a 1:1 ratio for 3 days. Proliferation was assessed by flow cytometry based on CFSE dilution and analyzed with FlowJo software to determine the suppressive capacity. Representative fitting curves are shown. (D) Suppression rates of Treg cells on Teff proliferation in the two groups (*n* = 3). Intergroup comparisons were analyzed by *t*‐test. (E–G) Serum levels of IL‐6 (E), IL‐17 (F), and IL‐10 (G) were quantified in the three groups by enzyme‐linked immunosorbent assay (ELISA) (*n* = 4). Intergroup comparisons were analyzed by ANOVA.  ^∗^ *p* < 0.05;  ^∗∗^ *p* < 0.01;  ^∗∗∗^ *p* < 0.001; MFI, mean fluorescence intensity.

To explore the functional importance of IL‐6 signaling in vivo, LMT‐28 was administered to EAP mice as a blocker of the IL‐6 receptor. Histological analysis demonstrated that LMT‐28 treatment markedly reduced lymphocytic infiltration in prostate tissues compared with untreated EAP mice (Figure 5F). Histological scoring demonstrated that LMT‐28 treatment significantly reduced prostate inflammation compared with untreated EAP mice (*p* < 0.01; Figure 5G). Pelvic pain responses were also significantly alleviated in LMT‐28‐treated mice (Figure 5H). Immunohistochemical staining further showed that STAT5a expression in prostate‐infiltrating lymphocytes was restored following LMT‐28 treatment (Figure 5I). Flow cytometric assessment showed that administration of LMT‐28 markedly lowered Th17 cell frequency in the spleen and prostate (*p* < 0.01; Figure 6A–C, F,G). Conversely, Treg cell frequency was significantly increased (*p* < 0.05; Figure 6D, E, H, I). Consistent with these findings, CTLA‐4 expression in Treg cells was significantly restored following LMT‐28 treatment (844.25 ± 24.83 vs. 495.00 ± 45.58, *p* < 0.05; Figure 7A,B). Functional suppression assays further demonstrated that Treg suppressive capacity was significantly increased (13.82% ± 2.59% vs. 4.49% ± 1.94%, *p* < 0.01; Figure 7C,D). Serum cytokine analysis showed that LMT‐28 treatment significantly reduced IL‐6 (4.12 ± 0.61 vs. 7.09 ± 2.36 pg/mL, *p* < 0.01) and IL‐17 (7.57 ± 0.77 vs. 10.05 ± 1.05 pg/mL, *p* < 0.05) levels while increasing IL‐10 (4.10 ± 0.48 vs. 2.73 ± 0.25 pg/mL, ) (Figure 7E–G).

## 4. Discussion

CP/CPPS is a multifactorial disorder characterized by persistent pelvic pain and lower urinary tract symptoms, yet its underlying immunological mechanisms remain incompletely understood. Increasing evidence suggests that immune dysregulation, particularly disruption of the Th17/Treg balance, contributes to CP/CPPS pathogenesis. In the current study, we observed that EAP mice exhibited a marked Th17/Treg imbalance accompanied by impaired Treg suppressive function. These alterations were associated with elevated serum IL‐6 levels. Furthermore, pharmacological inhibition of IL‐6 signaling partially restored Treg function, improved the Th17/Treg balance, and alleviated inflammatory responses in EAP mice. Together, these findings support an association between IL‐6–related immune dysregulation and prostatitis progression.

IL‐6, a cytokine with broad immunoinflammatory functions, plays an important role in a wide range of chronic inflammatory and autoimmune diseases, such as rheumatoid arthritis, systemic lupus erythematosus, and inflammatory bowel disease [16, 26, 27]. Increased IL‐6 levels are frequently correlated with greater disease severity, and therapeutic blockade of IL‐6 signaling has demonstrated clinical benefits. For instance, inhibition of IL‐6 receptor signaling significantly improves clinical activity in individuals with rheumatoid arthritis [28], and IL‐6 blockade reduces intestinal inflammation in experimental colitis models [29]. Consistent with these observations, our study revealed significantly increased IL‐6 levels in EAP mice. This increase was accompanied by a higher Th17/Treg ratio, with elevated Th17 cells and reduced Treg cells. Previous work has identified IL‐6 as a key regulator that promotes naïve CD4^+^ T‐cell differentiation toward the Th17 lineage while limiting Treg development [30–32]. Our in vitro differentiation assays yielded similar observations, suggesting that elevated IL‐6 levels may contribute to immune imbalance in EAP.

In addition to altered T‐cell differentiation, impaired Treg suppressive function represents another important feature of chronic inflammatory diseases. Treg dysfunction has also been observed in autoimmune disorders, including rheumatoid arthritis and systemic autoimmune diseases [33]. In this study, Treg cells isolated from EAP mice displayed significantly diminished suppressive function compared with their control counterparts. IL‐6 stimulation in vitro further reduced Treg suppressive capacity and decreased CTLA‐4 expression, a critical molecule involved in Treg‐mediated immunoregulation. Conversely, inhibition of IL‐6 signaling partially restored Treg function and reduced inflammatory responses in vivo. These findings indicate that elevated IL‐6 levels in the inflammatory microenvironment may contribute to Treg dysfunction during EAP. Mechanistically, our evidence suggests that IL‐6‐driven attenuation of STAT5a signaling may underlie the loss of Treg suppressive function. STAT5a serves as a central regulator of Treg lineage stability and functional maintenance. STAT5a signaling plays an essential role in preserving Foxp3 expression and sustaining the functional stability of Treg cells [22, 34]. In the present study, IL‐6 stimulation was linked to reduced STAT5a expression and phosphorylation in Treg cells. Moreover, pharmacological inhibition of STAT5a signaling markedly reduced Treg suppressive capacity, supporting a potential role of this pathway in regulating Treg function. Although our results point to a possible connection between IL‐6 signaling and STAT5a activity, the exact molecular basis underlying this interaction remains unclear and requires further investigation. This study has several limitations that warrant mention. First, the inflammatory microenvironment of CP/CPPS likely involves multiple cytokines and immune pathways beyond IL‐6. Other inflammatory mediators may also influence Treg stability and Th17 differentiation either independently or in combination with IL‐6 signaling. Second, although our findings suggest an association between IL‐6 signaling, STAT5a activity, and Treg dysfunction, additional mechanistic studies are required to further clarify these interactions. Future investigations using genetic models or pathway‐specific interventions may provide deeper insight into these regulatory mechanisms.

## 5. Conclusion

**Figure 8:**
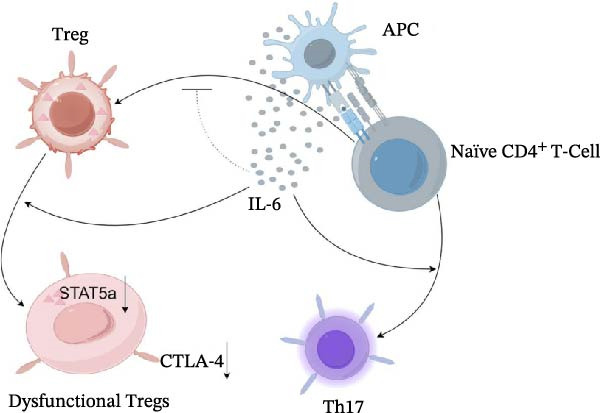
Hypothetical schematic model illustrating the potential mechanism by which IL‐6 contributes to Th17/Treg imbalance in EAP. Elevated IL‐6 may be associated with reduced STAT5a signaling in Treg cells, resulting in decreased CTLA‐4 expression, impaired Treg suppressive function, and enhanced Th17 differentiation.

In summary, our findings suggest an association between increased IL‐6 levels and impaired Treg cell suppressive function and Th17/Treg imbalance in EAP. Reduced STAT5a signaling may contribute to Treg dysfunction and decreased CTLA‐4 expression in this context. Pharmacological inhibition of IL‐6 signaling partially restored Treg function and alleviated inflammatory responses in EAP mice. The proposed mechanism derived from our findings is summarized schematically in Figure 8. These findings offer additional insight into the immunopathogenesis of CP/CPPS and highlight IL‐6–related signaling pathways as potential targets for future therapeutic strategies.

## Author Contributions

**Xianguo Chen**, **Jun Zhou**, and **Chaozhao Liang**: conception, design of this study. **Xianhong Liu**, **Xiaokang Bian**, and **Shanchuan Han**: collection, assembly of data. **Xianhong Liu** and **Boyang Li**: data analysis, interpretation. **Xianhong Liu**, **Boyang Li** and **Jian Song**: manuscript writing. All authors contributed to the article.

## Funding

This work was supported by the National Natural Science Foundation of China (Grants 81970597 and 82170787).

## Disclosure

All authors approved the submitted version.

## Ethics Statement

All animal procedures were performed in accordance with institutional requirements and were approved by the Animal Ethics Committee of Anhui Medical University (Approval Number LLSC20211521). The study was also carried out in line with the ARRIVE guidelines.

## Conflicts of Interest

The authors declare no conflicts of interest.

## Data Availability

The data generated and analyzed during the current study are available from the corresponding author upon reasonable request.
